# Evolving strategies for meningococcal vaccination in Europe: Overview and key determinants for current and future considerations

**DOI:** 10.1080/20477724.2021.1972663

**Published:** 2021-09-27

**Authors:** Federico Martinón-Torres, Muhamed-Kheir Taha, Markus Knuf, Victoria Abbing-Karahagopian, Michele Pellegrini, Rafik Bekkat-Berkani, Véronique Abitbol

**Affiliations:** a Genetics, Vaccines and Pediatric Infectious Diseases Research Group (GENVIP, Instituto De Investigación Sanitaria De Santiago and Universidad De Santiago De Compostela (Usc), Santiago de Compostela, Galicia, Spain; b Institut Pasteur, Invasive Bacterial Infections Unit, National Reference Centre for Meningococci and Haemophilus Influenza, Paris, France; c Klinik Für Kinder- Und Jugendmedizin, Worms, Germany and Pediatric Infectious Diseases, University Medicine, Mainz, Germany; d GSK, Amsterdam, The Netherlands; e GSK, Siena, Italy; f GSK, Rockville, MD, United States; g GSK, Rueil-Malmaison, France

**Keywords:** Invasive meningococcal disease, meningococcal vaccination, Europe, strategies, national immunization programme, MenACWY vaccination, MenB vaccination

## Abstract

Invasive meningococcal disease (IMD) is a life-threatening, unpredictable condition. Vaccines are available against 5 of the 6 meningococcal serogroups (Men) accounting for nearly all IMD cases worldwide; conjugate monovalent MenC, quadrivalent MenACWY, and protein-based MenB vaccines are commonly used. We provide a comprehensive overview of the evolution of meningococcal vaccination strategies employed in national immunization programmes (NIPs) and their impact on IMD incidence in Europe. A more in-depth description is given for several countries: the United Kingdom (UK), the Netherlands, Greece, Italy, and Ireland. We searched European health authorities’ websites and PubMed. Various vaccines and immunization schedules are used in 21 NIPs. Most countries implement MenC vaccination in infants, MenACWY in adolescents, and a growing number, MenB in infants. Only Malta has introduced MenACWY vaccination in infants, and several countries reimburse immunization of toddlers. The UK, Italy, Ireland, Malta, Andorra, and San Marino recommend MenB vaccination in infants and MenACWY vaccination in adolescents, targeting the most prevalent serogroups in the most impacted age groups. Main factors determining new vaccination strategies are fluctuating IMD epidemiology, ease of vaccine implementation, ability to induce herd protection, favorable benefit–risk balance, and acceptable cost-effectiveness. Since 1999, when the UK introduced MenC vaccination, the reduction in IMD incidence has been gradually enhanced as other countries adopted routine meningococcal vaccinations. Meningococcal vaccination strategies in each country are continually adapted to regional epidemiology and national healthcare priorities. Future strategies may include broader coverage vaccines when available (e.g., MenABCWY, MenACWY), depending on prevailing epidemiology.

## Background

Invasive meningococcal disease (IMD) is a life-threatening, unpredictable and uncommon condition, caused by the bacteria *Neisseria meningitidis*. Six of the 12 meningococcal serogroups, MenA, MenB, MenC, MenW, MenX, and MenY, account for nearly all IMD cases [1]. Their prevalence varies due to several factors, such as location, behavioral factors, economic setting, public health interventions in place to control and prevent the disease, and naturally occurring temporal fluctuations [2].

The annual incidence of IMD is relatively low, ranging worldwide from 0.01 to 3.6 cases per 100,000 persons over the last 2 decades, with the <1-year-old age group (infants) experiencing the highest incidence, followed by the 15–24-year-olds (adolescents and young adults), who are also the main carriers [1,3,4]. IMD has a rapid onset and high fatality rates even with optimal treatment (up to 20%, varying with the causing serogroup and age [5]). Long-term sequelae occur in 10–20% of survivors, with an important impact on their quality of life [6].

One of the important factors affecting IMD epidemiology are vaccination programmes. Plain polysaccharide vaccines have been available since the late 1960s [7], but their limitations have led to the development of conjugate vaccines, which (unlike plain polysaccharide vaccines) are able to induce proper, boostable immune responses in infants and long-term immunity, can provide a herd effect, and do not induce hypo-responsiveness upon repeated use [8]. Several conjugate monovalent (targeting MenC, MenA) and quadrivalent (MenACWY) vaccines and 2 protein-based vaccines providing broad protection against MenB strains are currently licensed for use [9]. One or more of these meningococcal vaccines are currently either recommended or included in national immunization programmes (NIPs) in several countries worldwide.

**Figure 1.**
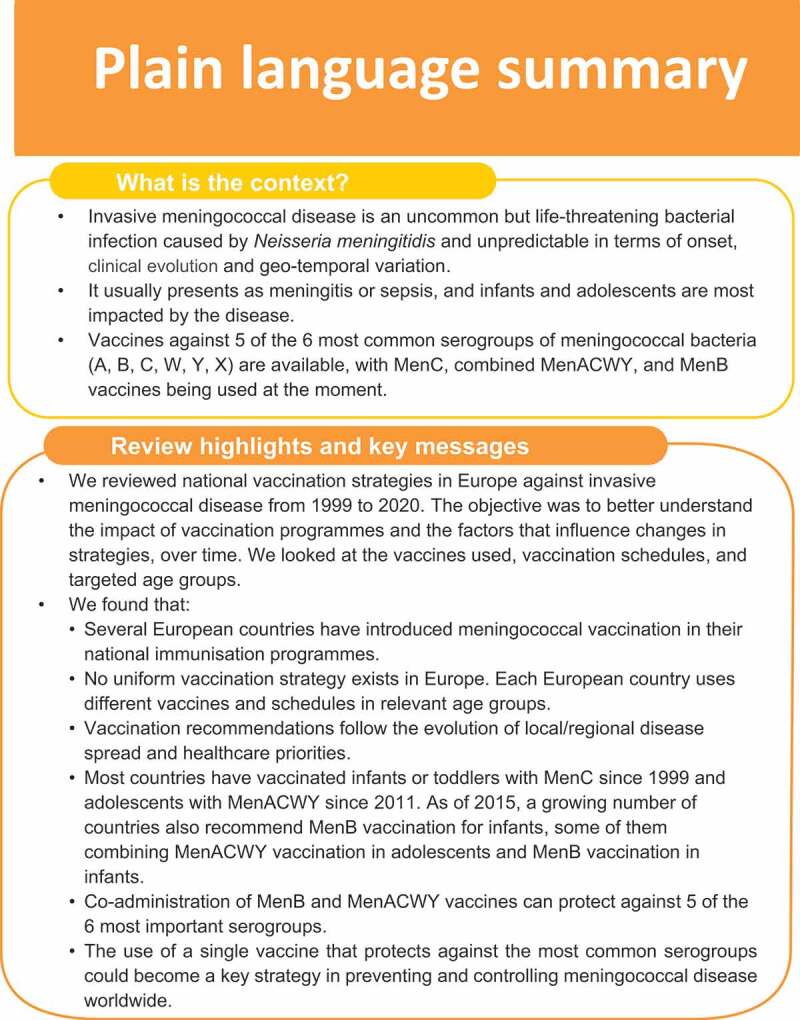
Plain language summary.

This review provides a comprehensive overview of the evolution of meningococcal vaccination strategies in terms of vaccines used, schedules and targeted age groups in European NIPs, and discusses determinants for implementation and their impact on meningococcal disease incidence. A plain language summary summarizing the key findings is presented in Figure 1.

## Methods

To identify past and current meningococcal vaccination schedules, we searched the websites of the European Centre for Disease Prevention and Control (ECDC) for countries in the European Union (EU) and the World Health Organization (WHO) for other countries in the WHO European region. National websites of health authorities (i.e., health ministries, government sites, etc.) were searched for additional details about changes in vaccination strategy and reasons behind them. Only recommendations for the general population were considered, with immunizations targeting at-risk groups being out of scope for this review. Content in all languages was searched, covering a period from 1999 (the year of the first introduction of a meningococcal vaccine in a European NIP) to August 2020.

In addition, relevant published papers were identified through a back-search strategy. A PubMed search was performed in August 2020, using ‘meningococcal vaccination’ as the search string and filters were applied to restrict results by publication type (review, systematic review), publication date (last 10 years), species (humans), and language (English). All search hits (n = 397) were initially screened by title and abstract, and subsequently by full text to identify reviews related to meningococcal disease epidemiology, vaccination strategies, impact of vaccination on disease incidence and other potentially relevant information. Selected data were extracted, together with relevant original references, with a focus on data pertaining to 5 countries (i.e., the United Kingdom [UK], the Netherlands, Greece, Italy, and Ireland) previously selected by the authors to present the variety of meningococcal vaccination strategies, to reflect the dynamics of these strategies, and cover milestones in the evolution of meningococcal vaccination in the WHO European region.

## Results

**Figure 2.**
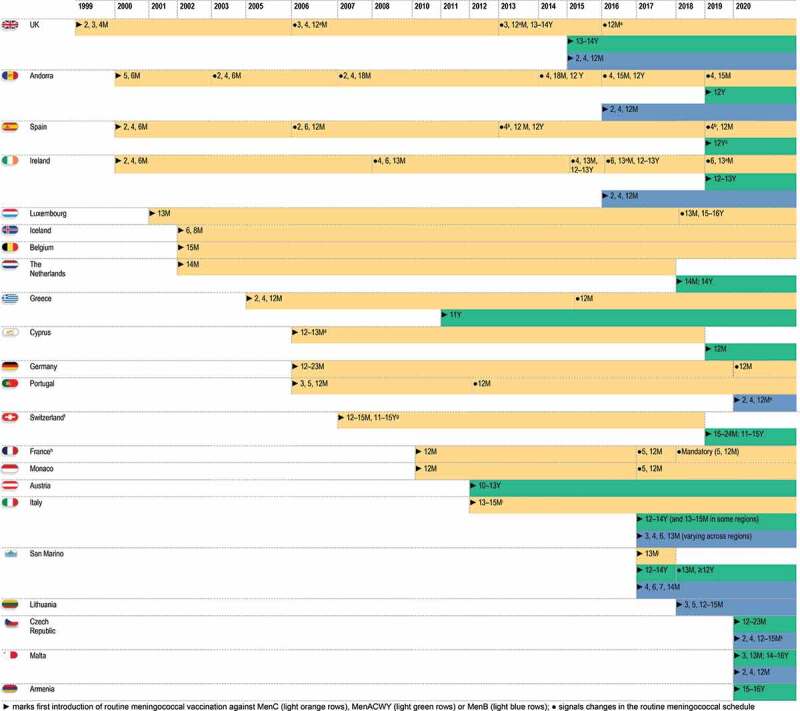
Evolution over time of meningococcal vaccination strategies in European countries.

An overview of different meningococcal vaccination strategies in the NIPs of 21 European countries throughout the period from 1999 to present, combining vaccine and schedule type and targeted age groups, is provided in Figure 2.

Notes:^a^ as Hib-MenC;^b^ depending on the vaccine used, an additional dose (at 2 months) can be administered;^c^ in 2 regions, a MenACWY dose is administered instead of the second dose of MenC vaccine at 12 months of age;^d^ two introduction phases (private and public [funded] sector); dates for the public sector are given;^e^ children born after the 1^st^ of January2021 are included in the programme;^f^ Liechtenstein follows the Swiss vaccination plan. MenC vaccination is recommended, but not part of the basic vaccination programme;^g^ part of the complementary vaccination schedule, which is fully reimbursed since 2006;^h^ in June 2021, the French National Authority for Health recommended that MenB vaccination is included in the national immunization programme;^i^ starting from 2005 in some regions in Italy;^j^ MenC vaccination was initially offered free of charge to all those requesting it by 31 March2017;^k^ MenB vaccination is free of charge if the first dose is administered before 6 months of age. References supporting the information in this figure are provided in **Supplement S1**.

### MenC vaccination

**Figure 3.**
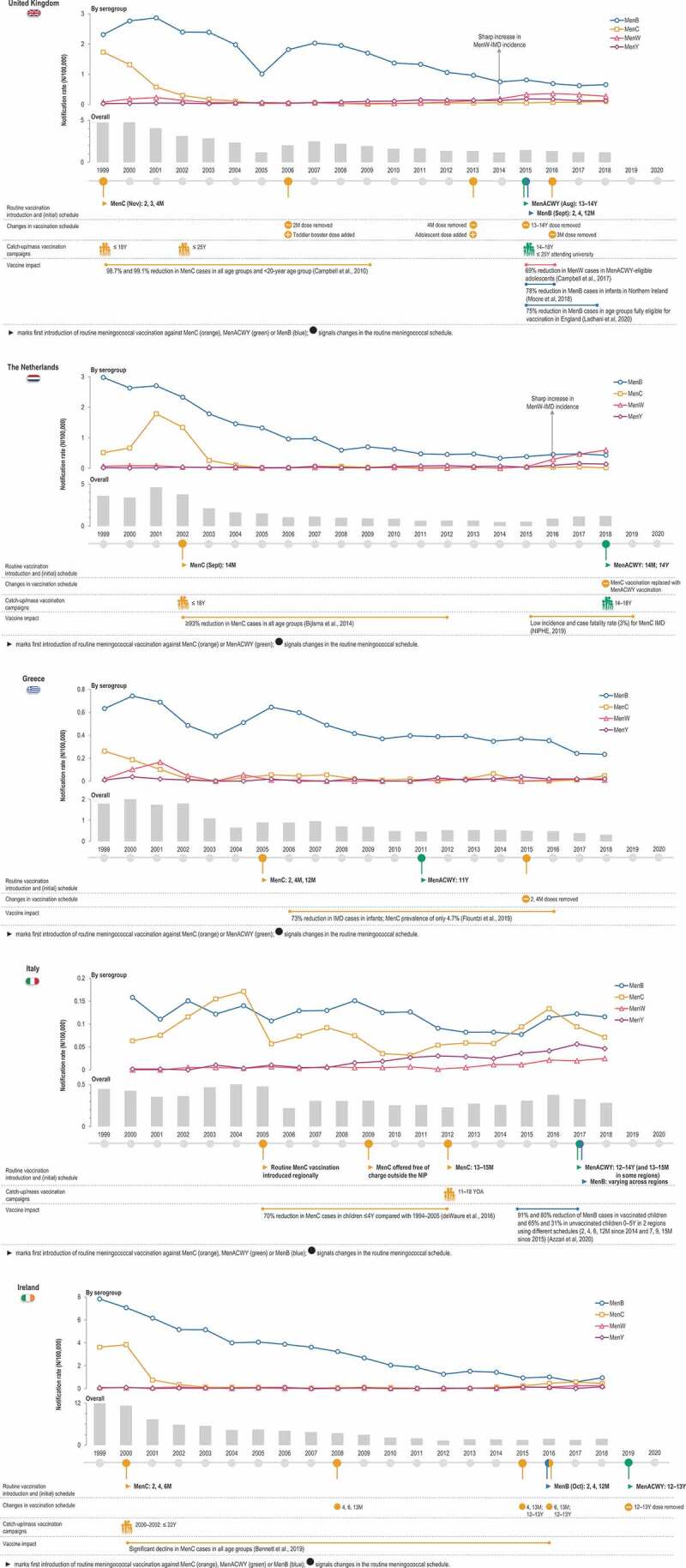
Evolution of serogroup-specific invasive meningococcal disease incidence and meningococcal vaccination strategies for 5 European countries.

IMD, invasive meningococcal disease; Men, meningococcal serogroup; M, months; N, number of cases; NIP, national immunization programme; Y, years. Note:Notification rates from the ECDC Surveillance Atlas [21].

In the UK, an increase of MenC infections was observed between 1994 and 1999, mainly due to the emergence of a hypervirulent ST11 strain, which caused severe symptoms and was associated with high case fatality rates in young children as well as adolescents [10]. To generate the immunogenicity and safety data needed for the licensure of MenC conjugate vaccines, a research programme was undertaken by the National Vaccine Evaluation Consortium in collaboration with MenC vaccine manufacturers, resulting in the accelerated licensure of MenC conjugate vaccines [10,11]. As such, the UK, as a pioneer in Europe, introduced MenC vaccination in the NIP in 1999, with a 3-dose schedule of MenC conjugate vaccine given to infants at 2, 3, 4 months of age without a booster dose (Figures 2, Figure 3). Additionally, a large-scale catch-up vaccination campaign through schools and general practitioners was rolled out, expanding the targeted age groups to all children and adolescents up to 18 years. An economic model of the MenC vaccination programme assessed that it was likely cost-effective in all age groups with high disease incidence. School-based vaccination programmes targeting children and adolescents aged 5–17 years with 1 dose were estimated to be more cost-effective than 3-dose infant vaccination by general practitioners because of lower delivery costs and fewer doses [12]. Because an increase in MenC disease was observed in young adults during the initial catch-up campaign, the campaign was further extended in January 2002 to all individuals <25 years of age [10,13] (Figure 3). The vaccine coverage in the UK NIP was high in those targeted by the initial vaccination programme (89% in infants and >85% in children up to 14 years old) and by the winter of 2000–2001, MenC-IMD incidence in these groups decreased by 80% [13]. In the following years, MenC vaccine schedules were further adjusted. The vaccination course was changed to a 2 + 1 dose schedule at 3 and 4 months of age with a booster dose at 12 months of age in September 2006, as adequate priming was shown in infants after 2 MenC doses in immunogenicity studies [10] and as evidence emerged of a loss of effectiveness in infants more than 1 year after the 2,4,6-month schedule [14,15]. It became apparent that the high success of the vaccination programme was not attributable to the direct protection of infants but instead to reductions in carriage in older children and adolescents targeted by the catch-up strategies, resulting in reduced transmission which in turn led to indirect (herd) protection in infants [14,15]. The booster dose at 12 months of age was thus added with the intention to improve long-term protection. Subsequently, long-term follow-up studies showed that higher antibody levels were observed in adolescence and early adulthood if MenC vaccination was received at >10 years rather than at <10 years of age [16]. This observation was the basis for another change in 2013, with removal of the MenC dose at 4 months of age and the addition of a booster dose in adolescents aged 13–15 years to sustain the herd protection afforded by the previous campaigns [17], while at the same time allowing the future introduction of MenB vaccination in infants [18]. More recently in 2015, given the emergence of MenW-IMD among adolescents, the adolescent MenC dose was replaced by a MenACWY dose to provide direct and indirect protection against all 4 serogroups [17]. The MenC dose at 3 months of age was also removed (2016), as the health authorities considered that the reduced pathogen transmission generated by the adolescent immunization would provide sufficient indirect protection in infants [17]. Currently, a single dose of a MenC-*Haemophilus influenzae* type b (Hib) vaccine is administered at 12 months of age [19,20]. A rapid drop in the incidence of MenC-IMD cases (by 98.7%) and in the number of MenC-related deaths was observed in all age groups within the first 2 years after the vaccination programme’s implementation [14]. Currently, the incidence of MenC-IMD remains low in the UK [21,22] (Figure 3). Evidence continues to accumulate to support the fact that direct and indirect protection against MenC-IMD has been afforded by the vaccination programme implemented in the UK [17].

In the Netherlands, a drastic increase in the incidence of MenC-IMD in 2001 (Figure 3) and the associated burden of disease prompted the inclusion of meningococcal vaccination in the NIP as a single dose schedule with consecutive catch-up-programme. A cost-effectiveness analysis evaluating direct and indirect costs over a time horizon of 77 years – indicating catch-up campaigns and routine vaccination at 14 months of age as the most favorable scenario – played a major role in this decision [23]. Starting in September 2002, a single dose of a MenC vaccine was offered to toddlers at 14 months of age, and catch-up campaigns targeting those between 14 months and 18 years old were implemented [24]. The decision to use a single dose in the second year of life was based on several considerations, such as the already high number of vaccinations administered during infancy and a better development of the immune system in toddlers, which could lead to enhanced antibody responses as compared to infants [25]. The impact of MenC vaccine introduction was seen almost immediately (in the same year) in the Netherlands, both in vaccinated and not vaccinated age groups [26]. By 2012, a ≥ 93% decline in cases was observed even in individuals not targeted by vaccination, demonstrating long-lasting herd protection, for at least 10 years from vaccine introduction [27]. In 2019, the incidence of MenC-IMD was lower than 0.1/100,000 persons, and the case fatality rate (estimated since 2015) was 3% [28] (Figure 3).

In Greece, a dramatic increase in IMD cases, particularly caused by MenC, was observed in the late 1990s [29]. This led to the frequent use (by 72% of pediatricians) of the MenC conjugate vaccine since 2001, the year of its introduction in the country, even in the absence of an official recommendation for administration in infants [30]. MenC vaccination in infants was eventually implemented in the NIP in 2005, contributing to the considerable reduction in the incidence of IMD in all age groups and especially in infants (by 73% from 2006 to 2016). During this period, MenC prevalence was only 4.7% [31]. However, an increase in MenC-IMD incidence was observed in 2018 (Figure 3).

Italy has also issued regional recommendations for MenC vaccination starting in 2005 and targeting 1-year-olds, as a consequence of a high incidence of MenC-IMD and associated mortality during 1999–2004. Starting in 2009–2010, the vaccine was offered free of charge throughout regions of Italy and introduction in the NIP was not until 2012, as a single dose in toddlers 13–15 months of age [32]. These measures led to a dramatic drop in MenC cases [32] (Figure 3). Despite these measures, a recent outbreak occurred in Tuscany during 2015–2016, mainly due to a hypervirulent ST-11 MenC strain, with cases even in vaccinated individuals [33]. This resulted in a swift reaction from regional health authorities implementing mass MenACWY/MenC vaccination campaigns [34]. This example underlines the need for continuous surveillance and re-adjustment of vaccination strategies to account for changes in epidemiology and compensate for waning immunity.

In Ireland, a considerable increase in the number of IMD cases was also recorded during the 1990s, reaching an incidence of 11.6/100,000 in the epidemiological year 1999/2001 [35]. Routine MenC vaccination has been implemented in Ireland’s NIP since 2000 [36], leading to a significant decline in the prevalence of this serogroup [21] and contributing to the overall decrease in the incidence of IMD (Figure 3) [35,37].

Drawing mostly from the UK experience, other countries have also introduced MenC vaccination in their NIP since the early (e.g., Belgium or Luxembourg), or the late 2000s (e.g., Germany or France). The Netherlands’ strategy was used as model for the implementation of MenC vaccination in Germany in 2006, according to a single-dose schedule and catch-up campaign. France also introduced MenC vaccination in 2010 as a single dose administered at 12 months of age, with a catch-up campaign in <25-year-olds, planned to continue until attaining herd immunity [38]. However, vaccine uptake in adolescents and young adults remained too low to achieve this aim and an increase in the incidence among infants was observed during 2011–2016, leading to the introduction of an additional dose at 5 months of age. This was followed in 2018 by the decision to include both doses in the mandatory vaccination strategy implemented in France. A decrease in MenC-IMD incidence among infants was observed since 2017 [38]. France is currently the only country in Europe implementing mandatory vaccination against MenC.

The age groups targeted for MenC vaccination differ, with some countries having recommendations for infants and toddlers only (between 4 and 23 months of age) and some also targeting 11–16-year-olds (Figure 2). Most countries also implemented catch-up vaccinations for older children and adolescents, up to 17- (Germany), 18- (Portugal), or 19-years-old (Iceland). In Spain, a single-dose MenC vaccination is currently offered to individuals of any age who have not been vaccinated by 18 years of age [39].

Following the use of MenC vaccines in Europe, a decline in the incidence of MenC disease was observed. However, no decreasing trend was noted for countries without MenC vaccination in their NIP [40].

### MenACWY vaccination

**Figure 4.**
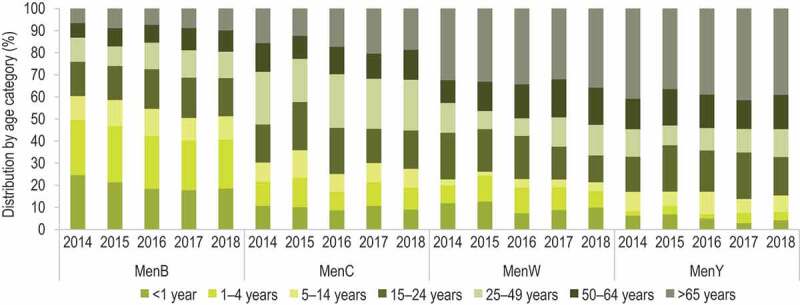
Distribution of reported serogroup-specific invasive meningococcal disease cases by age in European countries, 2014–2018

The incidence of MenW-IMD has displayed an increasing trend over the last decade in European countries, in particular in the Netherlands, Sweden, the UK, Switzerland [41], but also in Spain [42] and France [43]. In addition, while the prevalence of MenB and MenW invasive isolates in Europe differed substantially (~51% versus ~19%, respectively, in 2018), they accounted for a similar proportion of fatal IMD cases (36% versus 31%) [21], mainly due to the circulation of hypervirulent MenW strains belonging to the cc11 clonal complex [44,45], also observed for other serogroups (e.g., MenC in the Tuscany outbreak). An increase in the proportion of MenY-IMD has also occurred in certain European regions since 2010 [1,21,46]. MenW and MenY continue to be predominant in older adults (Figure 4), although a shift toward younger ages was observed after 2012 when compared with previous years.

Men, meningococcal serogroup. Note:Data from the ECDC Surveillance Atlas [21].

In UK, the adolescent MenC booster dose was changed to a MenACWY dose in 2015, in view of the availability of MenACWY conjugate vaccines and the increasing incidence of IMD caused by MenY and especially MenW, for which high case fatality ratios were observed [47]. This change was advised by the Joint Committee on Vaccination and Immunisation (JCVI), which underlined the added benefit of generating rapid herd protection against the circulating hypervirulent MenW strain through vaccinations at an age just before the one at which the highest carriage rates are observed [18]. Vaccine effectiveness and impact on meningococcal disease still need to be fully assessed. However, during the first year of an emergency adolescent MenACWY vaccination programme in England, there were 69% fewer MenW-IMD cases than predicted by trend analysis and no cases occurred in the first cohort to be vaccinated (adolescents who left school in 2015), despite low coverage (36.6%) [47]. Surveillance data show a steady decline in IMD notification rates due to MenY and MenW from 2016 to July 2020 [21,22].

Several other European countries have introduced MenACWY vaccination in their NIPs, targeting mainly adolescents. In most countries which had already implemented MenC vaccination (Spain, Andorra, Greece), the infant and/or toddler MenC dose was kept, but the adolescent MenC dose was replaced with MenACWY as in the UK. Some countries, such as Switzerland, Cyprus, and the Netherlands, adopted a different approach, with MenC vaccination being fully replaced by MenACWY over the last few years (Figure 2).

In the Netherlands, MenACWY vaccination was introduced in 2018 in direct response to an increased incidence of MenW-IMD and high case fatality rate (12% between 2015 and 2017), targeting adolescents 13–14 years old and replacing the MenC dose at 14 months of age. This strategy aimed to provide direct protection to the age group most affected by the increase in MenW-IMD, while at the same time reducing carriage among adolescents and boosting MenC responses, thus maintaining seroprotective levels needed for induction of herd effect. From 2019, MenACWY vaccination is being offered to all adolescents in the year they turn 14 years old. In the first half of 2019, a rapid decrease or stabilization of MenW-IMD incidence was observed in all age groups except ≥80-year-olds, with no cases recorded in vaccinated age groups [28].

Greece was among the countries which introduced routine MenACWY vaccination even without an observed increase in non-MenC-IMD incidence: an adolescent MenACWY booster dose at 11 years of age was included in the NIP in 2011 (Figure 3).

In Italy, MenACWY vaccination was introduced in 2017 as a single dose administered at 12–14 years of age [48], to afford sustained protection against the 4 serogroups during adolescence. As for Greece, no spike in MenW/MenY incidence had been observed in Italy, and the reported notification rates for all-cause IMD were lower than those in other European countries, with fluctuations over the last years (Figure 3) [21].

Ireland was among the countries in the EU that recently introduced MenACWY vaccination in their NIP. From September 2019, children in the first year of secondary school are being offered a MenACWY dose (Figures 2, Figure 3) [49], to boost immunity against MenC and provide additional protection against other meningococcal serogroups with an increasing trend in incidence since 2015.

The replacement of MenC with MenACWY vaccination at 14 months of age in the Netherlands, with the aim to broaden protection against IMD during childhood, made this country the first in Europe to implement MenACWY vaccination in toddlers, with Cyprus, Switzerland, Italy (in some regions), San Marino, and the Czech Republic being the only other ones. Malta is currently the only country in the EU implementing MenACWY vaccination in infants (Figure 2).

The impact of the various MenACWY vaccination strategies, including a potentially different effect on IMD incidence, is yet to be fully assessed.

### MenB vaccination

MenB continues to be the most prominent cause of IMD in Europe. In 2018, this serogroup accounted overall for approximately half of all IMD cases and was more prevalent among children 0–4 years of age compared with the other serogroups (Figure 4) [21]. A tendency for a slow but consistent decline in MenB incidence has been observed since 2013 [46], which can be attributed to natural fluctuations [50], as also observed worldwide [1,51]. Following the first licensure in 2013 of the 4-component MenB protein-based vaccine 4CMenB in the EU, several countries recommended vaccinations in high-risk groups and, in the last 5 years, as routine immunizations during infancy.

To address the consistently high proportion of MenB-IMD, the UK introduced 4CMenB in its NIP in September 2015, administered at 2, 4 and 12 months of age (Figure 2), with a single dose catch-up for infants aged 3–4 months. The implementation of a ‘reduced’ 2 + 1 schedule in the NIP instead of the 3 + 1 licensed in the EU was recommended by JCVI [52], based on cost-effectiveness. This recommendation made the UK the first country in the world to offer vaccination against MenB, hence covering the five most prevalent serogroups causing IMD through their NIP. During the 3 years after implementation, vaccination coverage reached >90% for the first two doses and 88% for the booster dose, resulting in a 75% reduction of MenB-IMD incidence (incidence rate ratio: 0.25; 95% confidence interval [CI]: 0.19–0.36) in the cohorts fully eligible for vaccination [53]. A reduction of 77.8% of MenB cases in the <1-year-olds was also reported in 2016 in Northern Ireland [54]. The impact of MenB vaccination in infants, at an early age, was substantial, even if MenB remains a prominent cause of IMD (Figure 3).

In Italy, 4CMenB was included in the NIP in 2017 [48]; the age at administration varies from one region to another, since the vaccine is not to be given at the same time with other routine immunizations (Figure 3). A national vaccination coverage of 38.6% has been reported for 2017, although in some regions >82% coverage was achieved [55]. A substantial decline in MenB cases was reported in two regions implementing different schedules of 4CMenB since 2014–2015 (2, 4, 6, 12 months in Tuscany vs. 7, 9, 15 months of age in Veneto) [56]. The relative case reduction calculated since introduction to 2017 was 91% and 80% in Tuscany and Veneto, respectively, in vaccinated children (mean vaccination coverage ≥80% in both regions), and 65% and 31% in unvaccinated children 0–5 years of age [56]. The higher relative case reduction observed in Tuscany for a vaccination coverage similar to that in Veneto, suggests that vaccination earlier in life leads to a greater impact on IMD incidence.

Ireland also recommends MenB immunizations, for all children born on or after 1 October 2016, using the same 3-dose schedule as in the UK, although no catch-up programme was implemented for older children [57]. Protection against 69.5% of MenB strains circulating prior to 4CMenB introduction was previously predicted [58], but data on the impact of vaccination is still expected.

MenB vaccination using 4CMenB has also been very recently implemented in the NIPs of other countries (Figure 2). In Portugal, a case-control study conducted during October 2014–March 2019 indicated an estimated vaccine effectiveness of 79% against MenB IMD, in individuals 2 months to 18 years of age [59]. These data supported the recent introduction of 4CMenB in the NIP, for all children born after January 1^st^, 2021 [60]. The expanding use of MenB vaccination in European NIPs and the continuously growing evidence accumulated from mass vaccination strategies are reinforcing the confidence in the prevention of MenB-caused IMD.

All countries currently implementing MenB routine immunization also have MenC/MenACWY vaccination in their NIP, except for Lithuania. In Malta, MenACWY and MenB NIP vaccinations target the same age group, with three doses of MenB vaccine (at 2, 4 and 12 months of age) alternating with MenACWY vaccinations (at 3 and 13 months of age); in addition, a booster MenACWY dose is recommended at 14–16 years of age (Figure 2). A recent recommendation to include infant MenB immunizations in the NIP (following a 2 + 1 schedule with 4CMenB) has also been issued in France [61], where only MenC vaccination is implemented at present. The recommendation is aimed at providing long-lasting individual protection to all infants, persisting up to 4 years of age based on data available so far, to make the vaccine accessible to vulnerable social categories, which are at higher risk of IMD, and reduce the impact of social inequity in health on the frequency and delay in management of infection. Other factors driving this recommendation included the good programmatic fit of 4CMenB in the NIP and the potential to protect against other meningococcal serogroups [61].

## Discussion

The worldwide epidemiology of IMD continues to fluctuate unpredictably and at a different pace. There is consistent and growing level of evidence that the implementation of meningococcal vaccination programmes has contributed to the decline of IMD incidence globally, supporting the prevention and control of IMD through vaccination across serogroups.

**Figure 5.**
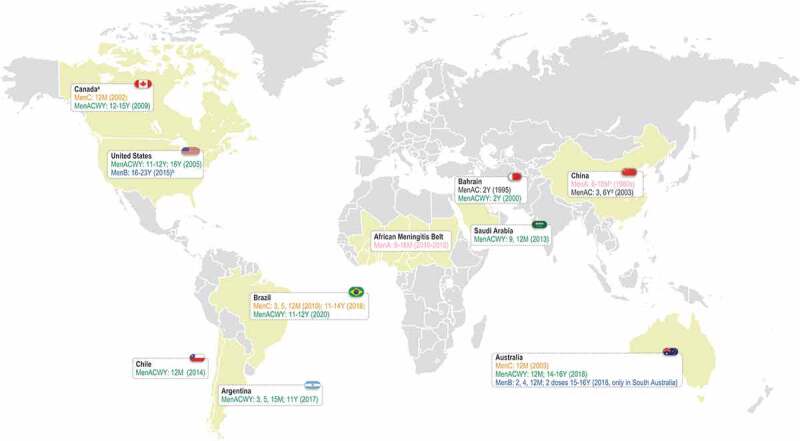
Introduction of routine meningococcal vaccination and current vaccination schedule in non-European countries

Outside of Europe, several countries currently recommend routine MenACWY vaccination (Figure 5), while others target at-risk individuals only or use it in response to outbreaks [62].

M, month (of age); Men, meningococcal serogroup; Y, year (of age). Notes:^a^ varies by provinces and territories, in terms of schedule and implementation date;^b^ on the basis of shared clinical decision-making;^c^ two doses, 3 months apart;^d^ if the child has not received the MenA polysaccharide vaccine between 2 and 4 years of age, he will receive the MenA plus MenC polysaccharide vaccine with a 3-year interval between the initial immunization and booster dose. References supporting the information in this figure are provided in **Supplement S1**.

Almost all countries in Europe introduced recommendations for meningococcal vaccination in high-risk populations as soon as vaccines were available and licensed, in view of the fatal nature of IMD. Over the last decades, more and more countries also implemented vaccinations in their NIP, initially targeting the age group with the highest risk and incidence of disease (infants). Gradually, policies expanded to also directly target adolescents, thus contributing to the prevention/control of IMD by reducing transmission within and from the age group with the highest carriage. Fluctuating IMD epidemiology is one of the most important determinants supporting the implementation and adoption of new vaccination programmes or the introduction of new vaccines. High incidence and mortality rates are obvious factors in establishing the age group prioritized for vaccination against this deadly disease. In Europe, IMD is subject to passive surveillance, with currently more than 30 countries reporting yearly notification rates to the ECDC. This allows continuous monitoring of IMD epidemiology in order to observe (and potentially anticipate) changes which would require an adaptation of vaccination strategies. In the selection of a certain meningococcal vaccine and schedule, the criteria considered by European countries are as follows: boostability and/or long-term persistence of immune response [11], effect on acquisition of carriage and ability to induce herd effect [20], in addition to a favorable benefit–risk balance and cost. Other health-related determinants are also considered by decision makers, such as cost-effectiveness of the vaccination programme, feasibility (in terms of current and long-term supply of vaccines), or the possibility for rapid and high uptake in the targeted population given the available resources for distribution and administration of the vaccine [63]. All these factors are critical determinants in the decision to implement new strategies for vaccination against IMD, and will vary from one country to another, often leading to diverse vaccination programmes and constituting a challenge to the use of a uniform recommendation across Europe and beyond.

Immunization against MenC in the first year of life is currently the most employed meningococcal vaccination strategy in Europe. With the very recent exception of Malta, no country has introduced MenACWY vaccination in infants so far, although several countries practice vaccination of toddlers. Adolescents remain an important target for vaccination, given that they are the main carriers and the need for sustained protection 10–15 years after priming against MenC. MenACWY vaccination in adolescence has the added benefit of a broader protection against other serogroups, including MenW, especially due to its transient upsurge in incidence in several countries. Moreover, the increasing proportions of atypical clinical presentation observed for MenW-IMD (gastrointestinal or upper respiratory tract symptoms) may delay rapid diagnosis and management, thus making prevention by vaccination an even more crucial strategy [64,65]. An increasing number of European countries have opted for the introduction of MenACWY in adolescents, while six (the UK, Italy, Ireland, Malta, Andorra and San Marino) have additionally implemented MenB vaccination in infants and MenC/MenACWY in the first 2 years of life. Currently, Malta is the only country in Europe to concomitantly implement MenB and MenACWY vaccinations in infants.

State-funded MenB vaccination in both infants and adolescents is to date implemented only in South Australia. In a state-wide cluster randomized trial in a large adolescent cohort 16–19 years of age, an overall reduction of MenB-IMD cases of 71% was observed within 2 years from 4CMenB vaccination of the 2017 cohort compared with 2003–2016 [66]. However, experience with other meningococcal vaccines seems to indicate that a combined vaccination strategy for 4CMenB would be the most successful in the control and prevention of MenB-IMD [67]. This would involve direct protection of infants, the age group with the highest IMD incidence, and older age groups by the administration of a catch-up or additional vaccination in adolescents. A modeling study assuming dynamic transmission, with separate variables for meningococcal carriage and IMD for MenB, MenACWY and ’Other’ mostly nonpathogenic serogroups showed that combined strategies with infant/adolescent 4CMenB and adolescent or toddler/adolescent MenACWY vaccination would result in the largest decrease in IMD cases [68].

Currently, at least two meningococcal vaccines are still required to prevent and control IMD caused by the five most prevalent serogroups. The co-administration of 4CMenB and MenACWY vaccines is already approved in the EU [69] and immune responses to vaccine antigens when co-administered have been shown to be comparable to those elicited by separate administration in infants [70]. However, future strategies may focus on the use of a single vaccine with broader serogroup coverage, which would allow for a better fit in the already complex NIP of most countries, and potentially improve compliance and costs by reducing the number of doses administered and visits to the clinic. Two MenABCWY candidate vaccines are currently under development. For one of these, several formulations including MenACWY-CRM and 4CMenB components have been shown to be immunogenic and well tolerated in adolescents and young adults [71] and to afford broad coverage of MenB strains [72,73]. Moreover, evidence supporting some extent of cross-protection against MenACWY and MenX strains continues to accumulate for 4CMenB [50,74–76]. In addition, 4CMenB may also induce partial cross-protection against *N. gonorrhea* [77]. Notably, the impact of 4CMenB vaccination in real-world settings has been shown in the UK, with a 69% reduction of MenW-IMD incidence in children fully eligible for vaccination (adjusted incidence rate ratio: 0.31; 95% CI: 0.20–0.67) and 98 MenW cases prevented directly by 4CMenB vaccination in children ≤12 years (eligible for 4CMenB but not MenACWY), during 2011/2012–2018/2019 [78]. While no impact on meningococcal carriage has been shown so far for 4CMenB [79], an important effect might still be achieved by direct protection against other serogroups [78,80] or by contributing to the prevention of *N. gonorrhea* in adolescents.

Therefore, while current epidemiological trends in Europe indicate that implementation of 4CMenB vaccination early in life (i.e., in infancy) may lead to the greatest reduction in IMD incidence, policy makers may already consider toddler and/or adolescent 4CMenB schedules, concomitantly with MenACWY vaccination. Real-world evidence from the UK and Italy where a combined 4CMenB and MenACWY strategy (albeit in different age groups) is already implemented and has demonstrated its value, as well as modeling studies [68], seem to support the use of vaccines that are concomitantly targeting all relevant meningococcal serogroups in different age groups. A pentavalent MenABCWY vaccine would enhance implementation of a broad meningococcal strategy and will most likely emulate the effect of the separate use of 4CMenB and MenACWY. A similar strategy is also considered in the African meningitis belt. The future use of a MenACWXY vaccine is believed to be the optimal approach to control IMD in this geographical area, following the reduction in MenA-IMD through mass vaccination, but in view of the continuous circulation of MenC, MenW and MenX [81].

This review’s main strength is that it presents the historical evolution of various meningococcal vaccination strategies according to the epidemiological evolution of IMD and their impact on disease incidence overtime in Europe and is not limited to current recommendations only. It also covers the implementation of meningococcal vaccinations against the most clinically relevant serogroups circulating in Europe, which can help inform stakeholders on different options for future strategies involving combinations of MenACWY and MenB vaccines. However, vaccination strategies are not described in detail for all countries. In addition, data on IMD incidence is only available for countries reporting to the ECDC and only up to 2018.

## Conclusion

Meningococcal vaccination strategies vary in Europe, with national health authorities recommending initially routine MenC vaccinations and, starting with 2011, also implementing MenACWY, MenB, or a combination of vaccination programmes. Countries are striving toward targeting the most relevant age groups with broad serogroup coverage for public health prevention and control of this invasive disease. Future vaccination strategies in each country will no doubt continue to be adapted to the particularities of regional IMD epidemiology and priorities. Nowadays, IMD may be considered a vaccine-preventable disease.

## Supplementary Material

Supplemental Material

## List of abbreviations

4CMenB, 4-component meningococcal serogroup B protein-based vaccine; ECDC, European Centre for Disease Prevention and Control; EU, European Union; Hib, *Haemophilus influenzae* type b; IMD, invasive meningococcal disease; JCVI, Joint Committee on Vaccination and Immunisation; Men, meningococcal serogroups; NIP, national immunization programmes; UK, United Kingdom.

## Data sharing statement

Data sharing not applicable to this article as no datasets were generated or analyzed during the current study.
