# Supplementary material for: Evolving strategies for meningococcal vaccination in Europe: Overview and key determinants for current and future considerations
Source: Pathog Glob Health. 2021 Sep 27;116(2):85–98. doi: 10.1080/20477724.2021.1972663 (PMC8933022; doi:10.1080/20477724.2021.1972663)
Supplement: Supplemental Material [file YPGH_A_1972663_SM5356.docx]

**References supporting the information presented in Figure 2**

| **Country** | **Reference** |
| --- | --- |
| UK | Public Health England. Chapter 22. Meningococcal- Immunisation against infection disease. 2016. [cited 2021 Jan 21]. Available from: <https://assets.publishing.service.gov.uk/government/uploads/system/uploads/attachment_data/file/554011/Green_Book_Chapter_22.pdf>.  Public Health England. Historical vaccine development and introduction of routine vaccine programmes in the UK. 2019. [cited 2021 Jan 21]. Available from: <https://assets.publishing.service.gov.uk/government/uploads/system/uploads/attachment_data/file/816174/Vaccine_Timeline_2019.pdf>. |
| Andorra | Government of Andorra, Department of Health. Vaccination plan. Infant vaccination. [cited July 4, 2020]. Available from: <https://www.salut.ad/departament-de-salut/pla-de-vacunacions>.  Government of Andorra. Decret del 10-02-2016 pel qual s’aprova la modificacio del Decret del 3 de febrer del 2016 d’actualitzacio del calendari de vacunacions del Pla de vacunacions sistematiques obligatories. Butlleti Oficila del Principat d'Andorra. Num. 12, 2016. [cited July 4, 2020]. Available from: https://www.bopa.ad/bopa/028012/Documents/GD20160212_13_47_42.pdf |
| Spain | Ministerio de Sanindad, Consumo y Bienestar Social, Spain. Recomendaciones de vacunación frente a la enfermedad meningocócica invasiva. Ponencia de Programa y Registro de Vacunaciones. 2019 March. [cited 2021 Feb 2]. Available from: <https://www.mscbs.gob.es/profesionales/saludPublica/prevPromocion/vacunaciones/docs/Recomendaciones_Vacunacion_Meningococo.pd>.  Ministerio de Sanidad, Consumo y Bienestar Social, Spain. Preguntas y respuestas sobre la vacunación frente a la meningitis. 2019. [cited 2021 Feb 2]. Available from: <https://www.mscbs.gob.es/profesionales/saludPublica/prevPromocion/vacunaciones/docs/Preguntas_respuestas_Vacunacion_frente_meningitis.pdf>.  European Centre for Disease Prevention and Control. Spain: recommended vaccinations. 2019 Dec. Available from: <https://vaccine-schedule.ecdc.europa.eu/Scheduler/ByCountry?SelectedCountryId=68&IncludeChildAgeGroup=true&IncludeChildAgeGroup=false&IncludeAdultAgeGroup=true&IncludeAdultAgeGroup=false>. |
| Ireland | Ireland's Health Service Executive, National Immunisation Office. School Program. 2020 Nov. [cited 2021 Feb 2]. Available from: <https://www.hse.ie/eng/health/immunisation/pubinfo/schoolprog/school.html>.  The National Immunisation Advisory Committee, Ireland. Chapter 13. Meningococcal infection. 2019 Oct. [cited 2021 Feb 2]. Available from: <https://www.hse.ie/eng/health/immunisation/hcpinfo/guidelines/chapter13.pdf>  Ireland's Health Service Executive, Health Protection Surveillance Centre. Bacterial Meningitis/Meningococcal Disease. 2019 Sept. [cited 2021 Feb 2]. Available from: <https://www.hpsc.ie/a-z/vaccinepreventable/bacterialmeningitis/factsheets/#:~:text=Group%20C%3A%20The%20meningococcal%20group,2%2C%204%20and%206%20months>. |
| Luxembourg | Le Conseil Supérieur des Maladies Infectieuses (CSMI), Louxembourg. Méningite et infections invasives à méningocoques. 2018 May. [cited 2021 Feb 2]. Available from: <https://sante.public.lu/fr/espace-professionnel/recommandations/conseil-maladies-infectieuses/meningite/>.  Le Conseil Supérieur des Maladies Infectieuses (CSMI), Louxembourg. Enquête de couverture vaccinale 2018 au Grand-Duché de Luxembourg. 2018. Available from: <https://sante.public.lu/fr/publications/e/enquete-couverture-vaccinale-lux-2018/enquete-couverture-vaccinale-2018.pdf>. |
| Iceland | Embætti Iandlæknis, Directorate of Health. Heilahimnubólga af völdum meningókokka C. 2003 Jan. [cited 2021 Feb 2]. Available from: <https://www.landlaeknir.is/um-embaettid/frettir/frett/item16816>.  European Centre for Disease Prevention and Control. Iceland: Recommended vaccinations. 2014 Apr. [cited 2021 Feb 2]. Available from: <https://vaccine-schedule.ecdc.europa.eu/Scheduler/ByCountry?SelectedCountryId=101&IncludeChildAgeGroup=true&IncludeAdultAgeGroup=true&SelectedVersionId=15>.  Gottfredsson M, Diggle MA, Lawrie DI, Erlensdóttir H, Hardardóttir H, Kristinsson KG, et al. Neisseria meningitidis sequence type and risk for death, Iceland. Emerg Infect Dis. 2006;12:1066-73. <http://dx.doi.org/10.3201/eid1207.051624>.  Thornórðardóttir A, Erlendsdóttir H, Sigurðardóttir B, Harðardóttir H, Reynisson IK, Gottfreðsson M, et al. Bacterial meningitis in adults in Iceland, 1995-2010. Scand J Infect Dis. 2014;46:354-60. <http://dx>.doi.org/10.3109/00365548.2014.880184.  Embætti Iandlæknis, Directorate of Health. Information about childhood vaccinations for parents and relatives. 2019 Nov. [cited 2021 Feb 2]. Available from: <https://www.landlaeknir.is/servlet/file/store93/item21251/LAN%2093074%20Bolusetningarbkl.enska_HQ.pdf>. |
| Belgium | European Centre for Disease Prevention and Control. Belgium: Recommended vaccinations. 2019 Sept. [cited 2021 Feb 2]. Available from: <https://vaccine-schedule.ecdc.europa.eu/Scheduler/ByCountry?SelectedCountryId=269&IncludeChildAgeGroup=true&IncludeChildAgeGroup=false&IncludeAdultAgeGroup=true&IncludeAdultAgeGroup=false>.  Superior Health Council, Belgium. Advisory 9485 Meningococcal vaccination. 2019 Jul. [cited 2021 Feb 2]. Available from: <https://www.health.belgium.be/sites/default/files/uploads/fields/fpshealth_theme_file/shc_9485_meningococcal_vaccination_2019_1.pdf>. |
| The Netherlands | European Centre for Disease Prevention and Control. Netherlands: recommended vaccinations. 2014 Apr. [cited 2021 Feb 2]. Available from: https://vaccine-schedule.ecdc.europa.eu/Scheduler/ByCountry?SelectedCountryId=163&IncludeChildAgeGroup=true&IncludeAdultAgeGroup=true&SelectedVersionId=25.  Bousema JC, Ruitenberg J. Need for Optimisation of Immunisation Strategies Targeting Invasive Meningococcal Disease in the Netherlands. Int J Health Policy Manag. 2015;4(11):757-761. doi: 10.15171/ijhpm.2015.168.  National Institute for Public Health and the Environment. The National Immunisation Programme in the Netherlands. Surveillance and developments in 2018-2019. 2019. [cited 2021 Feb 2]. Available from: <http://www.rivm.nl/bibliotheek/rapporten/2019-0193.pdf>.  Haverkate M, D'Ancona F, Giambi C, Johansen K, Lopalco PL, Cozza V, Appelgren E. Mandatory and recommended vaccination in the EU, Iceland and Norway: results of the VENICE 2010 survey on the ways of implementing national vaccination programmes. Euro Surveill. 2012;17(22). doi: 10.2807/ese.17.22.20183-en. |
| Greece | European Centre for Disease Prevention and Control. Greece: Recommended vaccinations. 2018 Jan. [cited 2021 Feb 2]. Available from: <https://vaccine-schedule.ecdc.europa.eu/Scheduler/ByCountry?SelectedCountryId=82&IncludeChildAgeGroup=true&IncludeChildAgeGroup=false&IncludeAdultAgeGroup=true&IncludeAdultAgeGroup=false>.  Official documents available from:  1. <https://www.moh.gov.gr/articles/health/dieythynsh-dhmosias-ygieinhs/emboliasmoi/ethniko-programma-emboliasmwn-epe-paidiwn-kai-efhbwn/6346-palaiotera-epe-paidiwn-kai-efhbwn?fdl=15148>  2. <https://www.moh.gov.gr/articles/health/dieythynsh-dhmosias-ygieinhs/emboliasmoi/ethniko-programma-emboliasmwn-epe-paidiwn-kai-efhbwn/6346-palaiotera-epe-paidiwn-kai-efhbwn?fdl=15148>  Tryfinopoulou K, Kesanopoulos K, Xirogianni A, Marmaras N, Papandreou A, Papaevangelou V, Tsolia M, Jasir A, Tzanakaki G. Meningococcal Carriage in Military Recruits and University Students during the Pre MenB Vaccination Era in Greece (2014-2015). PLoS One. 2016;11(12):e0167404. doi: 10.1371/journal.pone.0167404. |
| Cyprus | Koliou M, Kasapi D, Mazeri S, Maikanti P, Demetriou A, Skordi C, Agathocleous M, Tzanakaki G, Constantinou E. Epidemiology of invasive meningococcal disease in Cyprus 2004 to 2018. Euro Surveill. 2020;25(30). doi: 10.2807/1560-7917.es.2020.25.30.1900534.  European Centre for Disease Prevention and Control. Cyprus: recommended vaccinations. 2016 Aug. [cited 2021 Feb 2]. Available from: <https://vaccine-schedule.ecdc.europa.eu/Scheduler/ByCountry?SelectedCountryId=46&IncludeChildAgeGroup=true&IncludeChildAgeGroup=false&IncludeAdultAgeGroup=true&IncludeAdultAgeGroup=false>. |
| Monaco | Service Public du Gouvernement Princier du Monaco. Calendrier vaccinal pour les enfants et adolescents. 2018 Apr. [cited 2021 Feb 2]. Available from: <https://service-public-particuliers.gouv.mc/Social-sante-et-famille/Sante-publique/Prevention-et-depistage/Calendrier-vaccinal-pour-les-enfants-et-adolescents>. |
| Germany | Aktuelle Daten und Informationen zu Infektionskrankheiten und Public Health. Robert Koch Institute. Epidemiologisches Bulletin. 34, 2020. Available from: <https://www.rki.de/DE/Content/Infekt/EpidBull/Archiv/2020/Ausgaben/34_20.pdf?__blob=publicationFile>.  Statement of the German Standing Committee on Vaccination at the RKI. Recommendations of the Standing Committee on Vaccination  (STIKO) at the Robert Koch Institute – 2017/2018. EpidemiologischesBulletin. 34, 2017. Available from: <https://www.rki.de/EN/Content/infections/Vaccination/recommandations/34_2017_engl.pdf?__blob=publicationFile>.  Mitteilung der Ständigen Impfkommission am Robert Koch-Institut: Begründung der STIKO-Empfehlungen zur Impfung gegen Pneumokokken und Meningokokken vom Juli 2006. Epidemiologisches Bulletin. 31, 2006. Available from: [https://www.rki.de/DE/Content/Infekt/EpidBull/Archiv/2006/Ausgabenlinks/31_06.pdf%3F__blob%3DpublicationFile](https://www.rki.de/DE/Content/Infekt/EpidBull/Archiv/2006/Ausgabenlinks/31_06.pdf).  European Centre for Disease Prevention and Control. Germany: Recommended vaccinations. 2019 Aug. [cited 2021 Feb 2]. Available from: <https://vaccine-schedule.ecdc.europa.eu/Scheduler/ByCountry?SelectedCountryId=6&IncludeChildAgeGroup=true&IncludeChildAgeGroup=false&IncludeAdultAgeGroup=true&IncludeAdultAgeGroup=false>. |
| Portugal | Despacho n.º 12434/2019. Diário da República n.º 250/2019, Série II de 2019-12-30. 2019. Available from: <https://dre.pt/home/-/dre/127608823/details/maximized>.  Direção General da Saude. Programa Nacional de Vacinação 2020. 2020 Sept. [cited 2021 Feb 2]. Available from: <https://www.dgs.pt/normas-orientacoes-e-informacoes/normas-e-circulares-normativas/norma-n-0182020-de-27092020-pdf.aspx>.  European Centre for Disease Prevention and Control. Portugal: Recommended vaccinations. 2017 Jan. [cited 2021 Feb 2]. Available from: <https://vaccine-schedule.ecdc.europa.eu/Scheduler/ByCountry?SelectedCountryId=167&IncludeChildAgeGroup=true&IncludeChildAgeGroup=false&IncludeAdultAgeGroup=true&IncludeAdultAgeGroup=false>.  Rodrigues F, Morales-Aza B, Christensen H, Giles J, Ferreira M, Sikora P, Madanelo I, Lucidarme J, Januário L, Finn A. Oropharyngeal Carriage of Meningococcus in Portugal by Group and Clonal Complex 6 Years After Adolescent Vaccine Campaign. Pediatr Infect Dis J. 2015;34(11):1267-1269. doi: 10.1097/inf.0000000000000860.  Comissão de Vacinas da Sociedade de Infeciologia Pediátrica Sociedade Portuguesa de Pediatria. Recomendações sobre Vacinas Extra Programa Nacional de Vacinação 2020. 2020 Sept. [cited 2021 Feb 2]. Available from: <https://www.spp.pt/UserFiles/file/Seccao_Infecciologia/recomendacoes%20vacinas_sip_final_28set_2.pdf>. |
| Switzerland | INFOVAC. Mise à jour des recommandations de vaccination contre les méningocoques: introduction d’un vaccin quadrivalent conjugué. 2011 Aug. [cited 2021 Feb 2]. Available from: <https://www.infovac.ch/docs/public/neisseria/4-vaccin-quadrivalent-conjugue-2011.pdf>.  INFOVAC. Protection contre les maladies invasives à méningocoques : adaptation des recommandations de vaccination. 2018 Nov. [cited 2021 Feb 2]. Available from: <https://www.infovac.ch/docs/public/neisseria/protection-contre-les-maladies-invasives-a-meningocoques-adaptation-des-recommandations-de-vaccination.pdf>.  INFOVAC. Couverture vaccinale des enfants âgés de 2, 8 et 16 ans en Suisse, 2014–2016. 2018 Jun. [cited 2021 Feb 2]. Available from: <https://www.infovac.ch/docs/public/couverture_vaccinale_enfants_suisse_2014_2016.pdf>.  INFOVAC. Méningocoques. 2020 Nov. [cited 2021 Feb 2]. Available from: <https://www.infovac.ch/fr/les-vaccins/par-maladie/meningocoques>. |
| Liechtenstein | European Centre for Disease Prevention and Control. Liechtenstein: Recommended vaccinations. 2020 Feb. [cited 2021 Jul 6]. Available from: <https://vaccine-schedule.ecdc.europa.eu/Scheduler/ByCountry?SelectedCountryId=119&IncludeChildAgeGroup=true&IncludeAdultAgeGroup=true&SelectedVersionId=104>  Liechtenstein National Administration. Health promotion and prevention. Vaccinations. 2021. [cited 2021 Jul 6]. Available from: <https://www.serviceportal.li/de/privatpersonen/gesundheit-vorsorge-und-pflege/gesundheitsfoerderung-und-praevention/impfungen> |
| France | Taha MK, Gaudelus J, Deghmane AE, Caron F. Recent changes of invasive meningococcal disease in France: arguments to revise the vaccination strategy in view of those of other countries. Hum Vaccin Immunother. 2020:1-6. doi: 10.1080/21645515.2020.1729030. |
| Austria | European Centre for Disease Prevention and Control. Austria: Recommended vaccinations. 2020 Jan. [cited 2021 Feb 2]. Available from: <https://vaccine-schedule.ecdc.europa.eu/Scheduler/ByCountry?SelectedCountryId=18&IncludeChildAgeGroup=true&IncludeChildAgeGroup=false&IncludeAdultAgeGroup=true&IncludeAdultAgeGroup=false>.  Bundesministeriums für Soziales, Gesundheit, Pflege und Konsumentenschutz. Impfplan Österreich. 2021 Jan. [cited 2021 Feb 2]. Available from: <https://www.sozialministerium.at/Themen/Gesundheit/Impfen/Impfplan-%C3%96sterreich.html>. |
| Italy | Health Ministry Italy. Piano nazionale prevenzione vaccinale PNPV 2017-2019. 2017. [cited 2020 Jul 4]. Available from: <http://www.salute.gov.it/imgs/C_17_pubblicazioni_2571_allegato.pdf>. |
| San Marino | Instituto per la Sicurezza Sociale. Republica de San Marino. Vaccinazioni obbligatorie a San Marino. Riferimenti legislativi. [cited 2021 Feb 2]. Available from: <http://www.iss.sm/on-line/home/vaccini-e-vaccinazioni/vaccinazioni-obbligatorie-a-san-marino/docCatriferimenti-legislativi.49003166.1.50.1.html>: Delibera n.30 del 18-01-17 Somministrazione vaccinazioni raccomandate; Delibera n.38 del 21/03/18 Modifica calendario vaccinale per le vaccinazioni obbligatorie e raccomandate.  Instituto per la Sicurezza Sociale. Republica de San Marino. Meningite, ecco il programma di vaccinazioni dell’ISS. 2020 Jan. [cited 2021 Feb 2]. Available from: <http://www.iss.sm/on-line/home/articolo49013879.html>. |
| Lithuania | Minister of Health of the Republic of Lithuania. Order V-22 of August 2018 (2018-00344). [cited 2021 Feb 2]. Available from: <http://e-seimas.lrs.lt/portal/legalAct/lt/TAD/f4a925d0f50f11e79a1bc86190c2f01a?p>. |
| Czech Republic | Czech Republic. Law of 21 April 2020 amending Act No. 258/2000 Coll., on the protection of public health and amending certain related laws, as amended, and other related laws. 2020. [cited 2021 Feb 2]. Available from: <https://www.epravo.cz/_dataPublic/sbirky/2020/sb0073-2020.pdf>.  Recommendations of the Czech Vaccinology Society of the J. E. Purkyně Czech Medical Association for Vaccination against Invasive Meningococcal Disease. 2020 Jun. [cited 2021 Feb 2]. Available from: <http://www.szu.cz/uploads/IMO/2020_Recommendation_vaccination_IMD.pdf>. |
| Malta | National Immunisation Schedule. 2020 Jul. [cited 2021 Feb 2]. Available from <https://deputyprimeminister.gov.mt/en/phc/pchyhi/Pages/National-Immunisation-Schedule.aspx>. |
| Armenia | Republic of Armenia. Health Ministry. Regulatory documents. National Immunisation Programme. [cited 2021 Jul 6]. Available from: <http://armvaccine.am/am/national-program-of-immigration-to-armenia>. |

**References supporting the information presented in Figure 5**

| **Country/region** | **Reference** |
| --- | --- |
| Canada | An Advisory Committee Statement (ACS) National Advisory Committee on Immunization (NACI). Update on Quadrivalent Meningococcal Vaccines available in Canada. 2016 March. [cited 2021 Feb 2]. Available from: <https://www.canada.ca/en/public-health/services/publications/healthy-living/update-quadrivalent-meningococcal-vaccines-available-canada.html> |
| United States | Mbaeyi SA, Bozio CH, Duffy J, Rubin LG, Hariri S, Stephens DS, et al. Meningococcal Vaccination: Recommendations of the Advisory Committee on Immunization Practices, United States, 2020. MMWR Recomm Rep. 2020;69(9):1-41. doi: 10.15585/mmwr.rr6909a1. |
| Brazil | Departamento de Imunizações e Departamento de Infectologia. Calendário de Vacinação da SBP 2019. 2019 Aug. [cited 2021 Feb 2]. Available from: <https://www.sbp.com.br/fileadmin/user_upload/21273o-DocCient-Calendario_Vacinacao_2019.pdf> |
| Chile | Booy R, Gentile A, Nissen M, Whelan J, Abitbol V. Recent changes in the epidemiology of Neisseria meningitidis serogroup W across the world, current vaccination policy choices and possible future strategies. Hum Vaccin Immunother. 2019;15(2):470-480. doi: 10.1080/21645515.2018.1532248 |
| Argentina | Booy R, Gentile A, Nissen M, Whelan J, Abitbol V. Recent changes in the epidemiology of Neisseria meningitidis serogroup W across the world, current vaccination policy choices and possible future strategies. Hum Vaccin Immunother. 2019;15(2):470-480. doi: 10.1080/21645515.2018.1532248 |
| Bahrain | Saeed N, AlAnsari H, AlKhawaja S, Jawad JS, Nasser K, AlYousef E. Trend of bacterial meningitis in Bahrain from 1990 to 2013 and effect of introduction of new vaccines. East Mediterr Health J. 2016;22(3):175-182. doi: 10.26719/2016.22.3.175. |
| China | Li J, Shao Z, Liu G, Bai X, Borrow R, Chen M, et al. Meningococcal disease and control in China: Findings and updates from the Global Meningococcal Initiative (GMI). J Infect. 2018;76(5):429-437. doi: 10.1016/j.jinf.2018.01.007. |
| African Meningitis Belt | Alderson MR, LaForce FM, Sobanjo-Ter Meulen A, Hwang A, Preziosi MP, Klugman KP. Eliminating meningococcal epidemics from the African meningitis belt: The case for advanced prevention and control using next-generation meningococcal conjugate vaccines. J Infect Dis. 2019;220(220 Suppl 4):S274-S278. doi: 10.1093/infdis/jiz297. |
| Saudi Arabia | Booy R, Gentile A, Nissen M, Whelan J, Abitbol V. Recent changes in the epidemiology of Neisseria meningitidis serogroup W across the world, current vaccination policy choices and possible future strategies. Hum Vaccin Immunother. 2019;15(2):470-480. doi: 10.1080/21645515.2018.1532248. |
| Australia | Australian Government. Department of Health. National Immunisation Program – Meningococcal vaccination schedule from 1 July 2020 – Clinical advice for vaccination providers. 2020 Jul. [cited 2021 Feb 2]. Available from: <https://www.health.gov.au/resources/publications/national-immunisation-program-meningococcal-vaccination-schedule-from-1-july-2020-clinical-advice-for-vaccination-providers>.  Marshall HS, Lally N, Flood L, Phillips P. First statewide meningococcal B vaccine program in infants, children and adolescents: evidence for implementation in South Australia. Med J Aust. 2020;212(2):89-93. doi: 10.5694/mja2.50481. |
